# *Rhodopseudomonas pseudopalustris* Mitigates Alzheimer’s Disease-Related Pathology in *C. elegans* Models by Enhancing Antioxidant Defense Capacity and Immune Activity

**DOI:** 10.3390/antiox15070785

**Published:** 2026-06-24

**Authors:** Chuyu Song, Cui Deng, Tengyue Zhang, Wei Yao, Dapeng Li, Xiangming Wang

**Affiliations:** 1 Department of Neurobiology, School of Basic Medical Sciences, Laboratory for Clinical Medicine, Capital Medical University, Beijing 100069, China; 2 Department of Cell Biology, School of Basic Medical Sciences, Laboratory for Clinical Medicine, Capital Medical University, Beijing 100069, China; 3 Department of Anesthesiology, Xuanwu Hospital, Capital Medical University, 45 Changchun Street, Xicheng District, Beijing 100053, China

**Keywords:** Alzheimer’s disease, *R. pseudopalustris*, antioxidant defense

## Abstract

Alzheimer’s disease (AD) lacks effective disease-modifying therapeutics. Probiotics, promising neuroprotective candidates, exert benefits mainly by modulating gut-brain-axis (GBA) signaling. This study explored the anti-AD effects and mechanisms of *Rhodopseudomonas pseudopalustris* (*R. pse*). Using *Caenorhabditis elegans* (*C. elegans*) AD models, we evaluated AD-related phenotypes (learning deficits, paralysis) after *R. pse* administration, and performed genetic analysis and metabolomic profiling to clarify its regulatory pathways and metabolites. Mechanistically, *R. pse* significantly alleviated AD-related phenotype in *C. elegans*. It upregulated γ-glutamylcysteine synthetase (GCS-1) to enhance the glutathione (GSH)-dependent antioxidant defense. Knockout of the oxidation repair enzyme methionine sulfoxide reductase A-1 (MSRA-1) abolished the neuroprotective effects of *R. pse*, which was rescued by methionine. *R. pse* also activated activating transcription factor 7 (ATF-7)-mediated innate immunity and transforming growth factor β (TGF-β) signaling, with pantothenic acid as its functional metabolite. Collectively, *R. pse* is a potential anti-AD bacterium that mitigates AD model pathogenesis by enhancing the cellular antioxidant capacity, providing experimental evidence for bacteria-based AD interventions.

## 1. Introduction

Alzheimer’s disease (AD), the predominant subtype of neurodegenerative dementia, is characterized by intricate and multifaceted pathophysiology, encompassing the aberrant deposition of amyloid-beta (Aβ) plaques, the formation of neurofibrillary tangles comprising hyperphosphorylated tau protein, persistent chronic neuroinflammation, and exacerbated oxidative stress [1,2]. Currently, few disease-modifying therapies have gained clinical approval for AD. This unmet clinical urgency underscores an imperative demand for validated and reliable biomarkers to enable early diagnoses and precise pathological staging, which are fundamentally prerequisite for accelerating mechanistic research progress and optimizing standardized clinical management of AD [3,4]. Although emerging therapeutic avenues, including terahertz technology and nanoplatform-based systems, have been explored for AD intervention across multiple contemporary investigations [5,6,7], the existing treatments predominantly exert only symptomatic relief rather than reverse the disease process. Accordingly, it remains an urgent clinical imperative to develop innovative interventions capable of arresting or reversing progressive neurodegeneration, thereby achieving fundamental therapeutic efficacy against AD.

Growing understanding of the microbiota–gut–brain axis (MGBA) has confirmed that a stable gut microbiota balance plays a key role in maintaining normal neurological function. Increasing evidence shows that disrupted gut health worsens core AD pathologies such as abnormal Aβ buildup, elevated phosphorylated tau levels, chronic neuroinflammation and oxidative damage. For this reason, more research attention has focused on probiotics as a promising approach to modify underlying disease progression rather than merely relieve AD symptoms [8,9]. Accumulating evidence has validated the multifaceted protective properties of probiotics, rendering them a promising therapeutic candidate against the pathological progression of AD. Mechanistically, probiotics exert potent neuroprotective effects, mainly via regulating the microbiota–gut–brain axis. Such beneficial actions are achieved through multiple core pathways: suppressing neuroinflammation, modulating amyloid-β metabolic homeostasis, elevating the expression levels of neurotrophic factors, and maintaining the structural and functional integrity of the blood–brain barrier [10].

Mounting preclinical evidence accumulated from diverse experimental disease models strongly underscores the substantial therapeutic potential of probiotics as a multi-targeted and multifaceted intervention strategy against AD. Representative beneficial strains, including *Lactobacillus* species and *Bifidobacterium* species, have been consistently validated to effectively ameliorate AD-related behavioral impairments in cognitive and motor functions, while concurrently alleviating core neuropathological hallmarks that define the progressive neurodegenerative progression of AD [11]. Accumulating mechanistic investigations employing the *C. elegans* model have further broadened and enriched the existing spectrum of neuroprotective probiotic resources against AD pathogenesis. These comprehensive studies have solidly validated that a panel of additional functional bacterial strains, including *Lacticaseibacillus rhamnosus*, *Pediococcus parvulus*, *Bacillus subtilis*, and *Lactococcus laudensis*, are capable of exerting potent protective effects and effectively mitigating the emergence and progression of AD-like pathological alterations in vivo. Notably, the consistent and reproducible beneficial therapeutic outcomes observed across evolutionarily distant model organisms—encompassing mammalian systems alongside the simple *C. elegans*—substantially reinforces the biological rationality and reliability of probiotic-mediated anti-neurodegenerative efficacy [12,13,14].

*Rhodopseudomonas pseudopalustris* remains poorly characterized. In contrast, its closely related species *Rhodopseudomonas palustris*, a purple non-sulfur bacterium, harbors a single circular chromosome of approximately 5.4 Mbp. This well-studied species exhibits a strong adaptability to illuminated agricultural wastewaters, primarily attributed to its versatile metabolic capacity to utilize a broad spectrum of organic compounds derived from plant and animal residues. As a model microorganism, *R. palustris* has been extensively used in basic research investigating photophosphorylation, light-harvesting systems, nitrogen fixation, the anaerobic catabolism of aromatic compounds, and iron oxidation. Furthermore, its versatile metabolic network, capacity for light-driven adenosine triphosphate (ATP) synthesis, and exceptional persistence under growth-arrested conditions render it a promising chassis strain for biotechnological applications [15]. Nevertheless, to date, the functional roles and biological relevance of *R. palustris* and *R. pse* in AD pathogenesis remain entirely undocumented.

Here, we demonstrate that *R. pse* alleviates AD pathology by rescuing learning deficits and paralysis in *C. elegans*. *R. pse* enhances the expression of the enzyme GCS-1, and a mutation of MSRA-1 blocks the amelioration of the AD model by *R. pse*; however, methionine supplementation restores the MSRA-1 mutant phenotype. *R. pse* may enhance the innate immunity via an enhanced TGF-β signal. Metabolomic profiling identified pantothenic acid as a bioactive metabolite of *R. pse* that mediates its anti-AD effects.

## 2. Materials and Methods

### 2.1. C. elegans Strains and Culture

All *C. elegans* strains were cultured at 20 °C. The worms were grown on standard NGM (nematode growth medium) agar plates seeded with *Escherichia coli* (*E. coli*) OP50 [16]. The *C. elegans* strains used in this study are listed in Table 1.

### 2.2. Bacterial Strains and Growth Conditions

The bacterial strains utilized in this investigation were *E. coli* OP50 and *R. pse*. Glycerol stocks of these strains were stored at −80 °C. Isolated *R. pse* colonies were obtained by streaking the glycerol stocks onto NGM agar plates and incubating at 28 °C for 96 h. Single colonies of *R. pse* were then picked and inoculated into 1 mL of TSB (tryptic soy broth medium), followed by incubation at 28 °C for 96 h. The control *E. coli* OP50 was cultured under the same conditions as *R. pse*, except for a cultivation duration of 24 h. The dilution procedure was performed as follows: 500 μL of *R. pse* culture was added to 500 μL of OP50 culture to achieve a 1:1 (*v*/*v*) dilution. This bacterial mixture was used for all subsequent experiments.

### 2.3. Paralysis Assay

Paralysis assays were performed using the *C. elegans* strain GMC101 cultured at 20 °C. The GMC101 strain expresses the full-length human Aβ_1-42_ peptide in body wall muscle cells and exhibits a paralysis phenotype, which enables rapid phenotypic screening. Synchronized L4-stage worms (*n* = 4) were first transferred onto NGM plates seeded with either *E. coli* OP50 or OP50 + *R. pse* and incubated for 4 days. More than 30 healthy L4-stage worms were then selected and transferred to fresh NGM plates containing the same bacterial strain. The worms were subsequently subjected to heat shock at 25 °C for 24 h in a temperature-controlled incubator. Paralysis was scored 24 h post-treatment using a standardized assay: worms were classified as paralyzed if gentle head stimulation with a platinum wire elicited no response, failed to induce withdrawal within 3 s, or produced only slight head movements without body displacement. The paralysis rate was calculated as the percentage of paralyzed worms relative to the total number of worms assayed.

### 2.4. 16S rRNA Sequence

The 16S rRNA gene of *R. pse* was amplified via polymerase chain reaction (PCR) with the universal bacterial primers 341F (5′-ACTCCTACGGGAGGCAGCAG-3′) and 806R (5′-GGACTACCAGGGTATCTAAT-3′).

### 2.5. Non-Associative Learning Assay

Non-associative learning assays were performed using the *C. elegans* strains GRU101 and GRU102. The GRU102 strain drives the pan-neuronal expression of Aβ_1-42_, with GRU101 serving as its control strain, and it is suitable for investigating learning and memory abilities. Four L4-stage *C. elegans* were first transferred to NGM plates seeded with either OP50 or OP50 + *R. pse* and allowed to develop at 20 °C (consistent with the culture temperature in the paralysis assays). After 4 days, approximately 30 healthy L4 larvae were selected and transferred to fresh NGM plates containing the same bacterial food source. Upon reaching Day 1 of adulthood, the worms were subjected to a learning and memory assay. Each animal was first placed on an unseeded NGM plate for 30 s to acclimate to the environment. Subsequently, the tip of a sterile eyebrow hair was used to gently touch the worm’s head, triggering a backward retreat response. After the worm resumed forward locomotion, the tactile stimulus was repeated; this cycle was continued until the worm failed to exhibit a withdrawal response. The total number of touches recorded per animal was defined as the habituation learning index.

### 2.6. Food Choice Assay

Bacterial preference assays were performed using 3 cm diameter NGM agar plates. A reference line was drawn through the center of each plate to divide it into two equal halves. Subsequently, 2.5 µL aliquots of OP50 and *R. pse* bacterial suspensions, standardized to equivalent cell densities, were symmetrically spotted onto the two halves of the medium. More than 20 synchronized Day 1-stage GMC101 worms were carefully transferred to the exact center of each assay plate. Following incubation for the predetermined duration, the number of worms present at each bacterial spot (including those in direct contact with the bacterial lawn) was counted to quantify the bacterial preference.

### 2.7. Fecundity Assay

Individual L4-stage GMC101 (pre-cultured for one generation on plates seeded with the respective bacterial strains) worms (*n* = 3) were transferred to NGM plates seeded with either OP50 or OP50 + *R. pse* as the bacterial food source. Egg-laying was monitored daily starting from Day 1 of adulthood (24 h post-L4 stage). Each day, following quantification of the number of eggs laid per worm, the worms were transferred to fresh NGM plates containing the corresponding bacterial strain to ensure consistent food availability and eliminate the confounding effects of residual eggs or bacterial depletion. This daily transfer and egg-counting protocol was continued until the worms ceased egg production entirely. The total lifetime fecundity was calculated as the cumulative number of eggs laid per individual worm over the entire reproductive period.

### 2.8. Growth Rate Assay

Five Day-1 adult GMC101 worms were transferred to NGM plates seeded with either OP50 or OP50 + *R. pse* and allowed to lay eggs at 20 °C for 5 h. Following the egg-laying period, adult worms were carefully removed from the plates to avoid confounding effects of parental presence or bacterial depletion. After a 3-day incubation period at the same temperature (20 °C), the developmental stage of the resulting progeny was scored: each larva was categorized as either ≥L4 stage (i.e., L4 larva or young adult) or <L4 stage (i.e., L1–L3 larva). The developmental rate was calculated as the percentage of progenies that reached the ≥L4 stage relative to the total number of viable progenies assayed.

### 2.9. Motor Ability Assay

The locomotor capacity was assessed in GMC101 and N2 progenies reared on NGM plates seeded with either OP50 or OP50 + *R. pse*. Briefly, four L4-stage larvae were initially transferred to freshly seeded NGM plates and cultured at 20 °C. After 4 days, approximately 25 healthy L4-stage offspring were selected and transferred to fresh NGM plates containing the same bacterial food source. On Day 1 of adulthood, individual worms were gently placed in 100 µL droplets of M9 buffer on glass slides and allowed 30 s to acclimate to the liquid environment. The number of complete sinusoidal body waves (full oscillations) executed by each worm during the subsequent 30 s was quantified; partial head or tail movements that did not constitute a full body wave were excluded from the count.

### 2.10. Heat Stress Assay

Four L4-stage *C. elegans* larvae were transferred to NGM plates seeded with either OP50 or OP50 + *R. pse*. and cultured at 20 °C. After 4 days, at least 30 healthy L4-stage progenies were selected and transferred to fresh NGM plates containing the same bacterial food source, followed by an additional 24 h of incubation at 30 °C for GMC101. N2 wild-type animals were exposed to 35 °C for 4 h at the Day 1 adult stage (strain-specific heat stress conditions). Following the respective heat treatments, the survival rates were quantified as the number of live worms relative to the total number of worms, and the data were statistically analyzed to assess the strain-specific thermotolerance.

### 2.11. R. pse Functional Part Assay

Single colonies of OP50 or *R. pse* were inoculated into 15 mL of TSB medium and cultured at 28 °C for 24 h and 96 h, respectively. The bacterial cultures were then centrifuged at 13,000 rpm for 5 min. The resulting bacterial pellets were retained, while the supernatants were re-centrifuged and filter-sterilized through a 0.22 µm membrane filter. For the OP50-pellet group, the OP50 pellet was resuspended in 500 µL of ddH_2_O; 50 µL of this suspension was mixed with 150 µL of sterile *R. pse* supernatant, and 60 µL of the mixture was spread onto NGM plates. For the heat-inactivated *R. pse*-pellet group, the *R. pse* pellet was autoclaved (121 °C, 20 min), then resuspended in 500 µL of ddH_2_O; 150 µL of the inactivated pellet suspension was combined with 50 µL of OP50 suspension, and the 60 µL mixture was plated onto NGM plates.

### 2.12. Fluorescence Microscopy and Visualization Assay

A 3% (*w*/*v*) agarose solution was prepared by microwave-heating until completely dissolved, then briefly cooled to approximately 50 °C. A small volume of molten agarose was dispensed onto a glass slide using a weighing spatula, and a second glass slide was placed on top to press the solution into a uniform pad (~1 mm in thickness). After 10 s of solidification, the upper slide was carefully removed to expose the agarose pad. A 4× working solution of levamisole was prepared by diluting a 10× stock solution (1 mg/mL) in ddH_2_O. A 2.5 μL aliquot of this anesthetic solution was pipetted onto the center of the agarose pad. Worms at the appropriate developmental stage were picked using a platinum wire pick, transferred to the anesthetic-treated agarose pad, and fully immersed in the solution. A coverslip was then gently placed over the sample to ensure an even distribution of the worms and solution. Fluorescence imaging was performed using a Zeiss Imager M2 microscope (Carl Zeiss Microscopy GmbH, Jena, Germany) equipped with filter sets 20 (Rhodamine) and 38 (Endow GFP). *wyIs50120* strain fluorescence imaging was performed using a Zeiss LSM 980 confocal microscope with Airyscan 2 (Carl Zeiss Microscopy GmbH, Jena, Germany). The acquired images were analyzed using the ImageJ software (Version: 2.14.0/1.54g) to quantify the fluorescence intensity.

### 2.13. Metabolomic Sample Preparation of R. pse

Single colonies of OP50 or *R. pse* were inoculated into 40 mL of TSB medium and incubated at 28 °C for 24 h and 96 h, respectively. Bacterial cultures were centrifuged at 13,000 rpm for 5 min; the supernatant was discarded, and centrifugation was repeated under identical conditions. The resulting bacterial pellet was resuspended, washed with ddH_2_O, and centrifuged again. This washing procedure was repeated a total of three times to remove residual medium components. The final bacterial pellet was stored at −80 °C until the analysis.

### 2.14. Metabolome Analysis of R. pse

The final dataset, containing the information of the feature number, sample name and normalized feature area, was imported to the SIMCA18.0.1 software package (Sartorius Stedim Data Analytics AB, Umea, Sweden) for a multivariate analysis. The data were scaled and logarithmically transformed to minimize the impact of both noise and high variance of the variables. After these transformations, a PCA (principal component analysis), an unsupervised analysis that reduces the dimension of the data, was carried out to visualize the distribution and the grouping of the samples. The 95% confidence interval in the PCA score plot was used as the threshold to identify potential outliers in the dataset.

In order to visualize group separation and find significantly changed metabolites, a supervised orthogonal projections to latent structures discriminate analysis (OPLS-DA) was applied. Then, 7-fold cross-validation was performed to calculate the value of R2 and Q2. R2 indicates how well the variation of a variable is explained and Q2 means how well a variable can be predicted. To check the robustness and predictive ability of the OPLS-DA model, 200 times permutations were further conducted. Afterward, the R2 and Q2 intercept values were obtained. Here, the intercept value of Q2 represents the robustness of the model, the risk of overfitting and the reliability of the model; the smaller the better.

Furthermore, the value of variable importance in the projection (VIP) of the first principal component in the OPLS-DA analysis was obtained. It summarizes the contribution of each variable to the model. The metabolites with a VIP > 1 and *p* < 0.05 were considered as significantly changed metabolites. In addition, commercial databases, including KEGG and MetaboAnalyst, were used for the pathway enrichment analysis.

### 2.15. Drug Treatment

Pantothenic acid was dissolved in DMSO to prepare a 100 mM stock solution, which was stored at −20 °C until use. Prior to the experiment, the stock solution was diluted with liquid OP50 culture medium to a 10 μM working concentration. For plate preparation, 60 μL of the diluted mixture was evenly spread onto each NGM plate.

### 2.16. Statistical Analysis

All statistical analyses were performed using the GraphPad Prism 9.0 software. For comparisons between two independent groups, two-tailed unpaired *t*-tests were utilized to evaluate the statistical significance of differences. For comparisons involving three or more groups, a one-way analysis of variance (one-way ANOVA) was employed to detect significant differences among groups [17]. Tukey’s multiple comparisons test was applied to correct for multiple comparisons following the one-way ANOVA. All statistical analyses were conducted with a conventional significance threshold of α = 0.05, where a *p*-value < 0.05 was considered statistically significant. Statistically significant results in all figures are denoted using standardized graphical notations: * *p* < 0.05, ** *p* < 0.01, *** *p* < 0.001, and **** *p* < 0.0001. Non-significant results (*p* > 0.05) are marked as “ns” (not significant).

## 3. Results

### 3.1. R. pse Mitigates Pathological Deficits of the Aβ-Mediated AD Model

**Figure 1:**
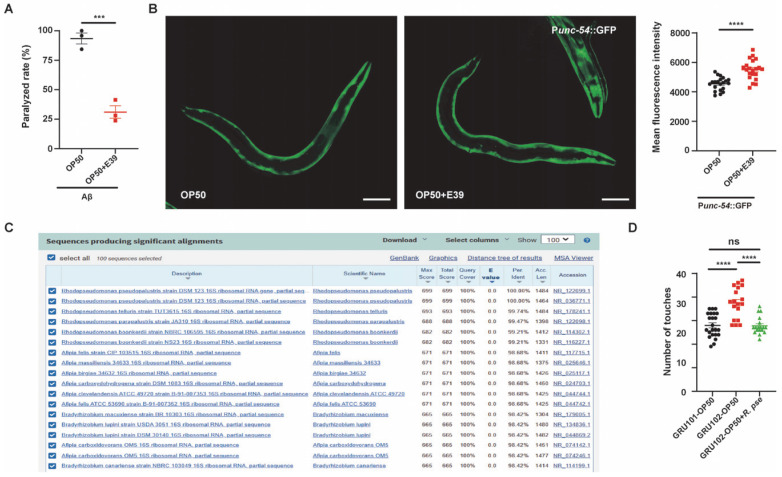
*R. pse* mitigates pathological deficits of the Aβ-mediated AD model. (**A**) The paralysis rate of the Aβ AD model treated with the control OP50 or OP50 + E39. Two-tailed unpaired Student’s *t*-test, three independent biological replicates. *** *p* < 0.001. (**B**) Representative fluorescence images and quantitative analysis of fluorescence intensity of P*unc-54*::GFP treated with OP50 or OP50 + E39. Scale bar = 100 μm. Data are the mean ± SEM; Two-tailed unpaired Student’s *t*-test. *n* = 21, **** *p* <0.0001. (**C**) BLAST-based taxonomic identification of strain E39 via 16S rRNA gene sequencing. (**D**) Scatter plot displaying number of touches in control GRU101 treated with OP50, AD model GRU102 treated with OP50 or OP50 + *R. pse*. Data are the mean ± SEM; one-way ANOVA followed by Tukey’s post hoc test, *n* = 18–23. **** *p* < 0.0001; ns, not significant (*p* > 0.05).

The expression of Aβ_1-42_ within the body wall muscle cells of *C. elegans* successfully generated the transgenic strain GMC101 (hereafter referred to simply as Aβ). This genetic manipulation markedly promoted the extensive formation and accumulation of toxic Aβ oligomers and aggregates throughout the tissues. These progressive pathological alterations subsequently triggered the onset of severe paralytic phenotypes in *C. elegans* [18]. By exploiting this well-characterized paralytic phenotype, we identified that E39, a bacterial contaminant isolated from routine laboratory *C. elegans* culture plates, markedly alleviated the paralysis severity in the Aβ-expressing AD model (Figure 1A). Given that Aβ expression in this transgenic strain was driven by the muscle-specific *unc-54* promoter, we further utilized a P*unc-54*::GFP reporter strain to verify whether the E39 treatment interfered with *unc-54* promoter activity. The quantitative fluorescence analysis revealed that exposure to E39 resulted in the enhancement of GFP intensity relative to the OP50-fed control group, rather than a decrease (Figure 1B). Collectively, these results demonstrated that E39 mitigated Aβ-associated pathological deficits independent of *unc-54* transcriptional regulation, thereby suggesting a direct modulatory effect on Aβ-mediated toxicity rather than altered promoter-driven expression. To determine the taxonomic classification of strain E39, 16S rRNA gene sequencing was conducted. A BLAST 2.17.0 (Basic Local Alignment Search Tool, National Center for Biotechnology Information) analysis suggested that strain E39 was most closely related to *Rhodopseudomonas pseudopalustris* (Figure 1C).

Cognitive dysfunction, exemplified by an impaired learning and memory capacity, constitutes the core pathological hallmark of AD progression. To systematically characterize the protective efficacy of *R. pse* against AD-associated cognitive deterioration, we adopted a classic experimental paradigm evaluating non-associative learning behaviors in *C. elegans*. This assessment relies on habituation traits, a conserved behavioral phenomenon in which worms exhibit a gradual reduction in responsiveness following repeated exposure to sensory stimuli (head touch), serving as a reliable readout for evaluating the fundamental learning and memory performance in vivo [19]. By employing the pan-neuronal Aβ-expressing transgenic strain GRU102 as the AD pathological model, we assessed the cognitive performance via the habituation paradigm. Relative to the control wild-type background strain GRU101, the GRU102 AD model exhibited markedly elevated touch stimulation requirements to reach effective habituation, reflecting robust learning dysfunction driven by neuronal Aβ cytotoxicity across the nervous system. Further functional validation illustrated that treatment with *R. pse* prominently lowered the number of mechanical touch stimuli necessary for successful habituation in GRU102 individuals (Figure 1D), suggesting a strong protective capacity of *R. pse* in rescuing Aβ-elicited physiological learning behaviors in vivo.

### 3.2. Effects of R. pse on WT and AD Models

**Figure 2:**
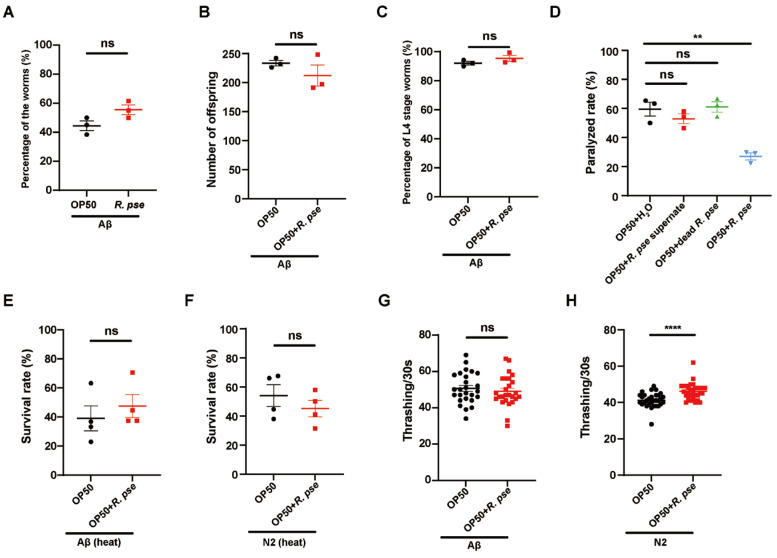
Effects of *R. pse* on WT and AD models. (**A**) Scatter plot showing food preference of the Aβ AD model to *R. pse* and OP50. Two-tailed unpaired Student’s *t*-test, three independent biological replicates. ns, not significant (*p* > 0.05). (**B**) Scatter plot showing number of offspring of the Aβ AD model treated with OP50 or OP50 + *R. pse*. Two-tailed unpaired Student’s *t*-test, three independent biological replicates. ns, not significant (*p* > 0.05). (**C**) Scatter plot showing the growth rate of the Aβ AD model treated with OP50 or OP50 + *R. pse*. Two-tailed unpaired Student’s *t*-test, three independent biological replicates. ns, not significant (*p* > 0.05). (**D**) The paralysis rate of AD models treated with OP50, OP50 + *R. pse*, OP50 + supernatant of *R. pse*, or OP50+ dead *R. pse* (autoclaving). One-way ANOVA followed by Tukey’s post hoc test, three independent biological replicates. ** *p* < 0.01; ns, not significant (*p* > 0.05). (**E**) Scatter plot showing heat resistance of the Aβ AD model treated with OP50 or OP50 + *R. pse*. Two-tailed unpaired Student’s *t*-test, four independent biological replicates. ns, not significant (*p* > 0.05). (**F**) Scatter plot showing heat resistance of N2 treated with OP50 or OP50 + *R. pse*. Two-tailed unpaired Student’s *t*-test, four independent biological replicates. ns, not significant (*p* > 0.05). (**G**) Scatter plot showing motility of Aβ AD model treated with OP50 or OP50 + *R. pse*. Data are the mean ± SEM; two-tailed unpaired Student’s *t*-test. *n* = 26. ns, not significant (*p* > 0.05). (**H**) Scatter plot showing motility of N2 treated with OP50 or OP50 + *R. pse*. Data are the mean ± SEM; two-tailed unpaired Student’s *t*-test. *n* = 30–31. **** *p* < 0.0001.

To characterize *R. pse* and evaluate its potential role in alleviating AD-related pathology, we systematically compared its biological properties with those of the control strain *E. coli* OP50. First, *R. pse* displayed a comparable colony-forming ability (9.5 × 10^8^ CFU) relative to OP50 (1.91 × 10^9^ CFU). Second, in food-preference assays, the worms showed no significant preference between *R. pse* and the standard food source OP50 (Figure 2A). Third, feeding of *R. pse* did not alter the brood size in the Aβ-based AD model (Figure 2B), indicating no detrimental impacts on reproductive fitness. Fourth, developmental progression from eggs to the fourth larval stage (L4) was unaffected by *R. pse* administration (Figure 2C), signifying normal growth kinetics within the AD-relevant genetic background.

To investigate whether the protective effect of *R. pse* relies on bacterial viability, we administered autoclave-inactivated *R. pse* to the Aβ-expressing AD model. We observed that the protective effect was abolished upon autoclaving (Figure 2D). To further determine whether the secreted factors alone are sufficient for protection, we supplemented the diet (OP50) with the cell-free culture supernatant of *R. pse*. Treatment with the supernatant failed to delay paralysis in the Aβ AD model (Figure 2D), suggesting that the bioactive constituents are not secreted into the extracellular milieu and likely depend on intact or metabolically active bacterial cells.

To further explore the potential of *R. pse* in enhancing stress resistance in *C. elegans*, we assessed its effect under heat stress and found that, compared with OP50 controls, *R. pse* could not improve the survival rate of either wild-type N2 or Aβ-based AD model worms under heat exposure (Figure 2E,F). Moreover, to determine whether the beneficial effects of *R. pse* extend beyond neuroprotection, we evaluated its impact on locomotor behavior and observed that *R. pse* administration enhanced thrashing activity in wild-type N2 worms in the M9 buffer, whereas no significant improvement in locomotion was detected in the Aβ AD model relative to OP50 controls (Figure 2G,H).

### 3.3. R. pse Mitigates Pathology in the Aβ AD Model by Anti-Oxidative Stress

**Figure 3:**
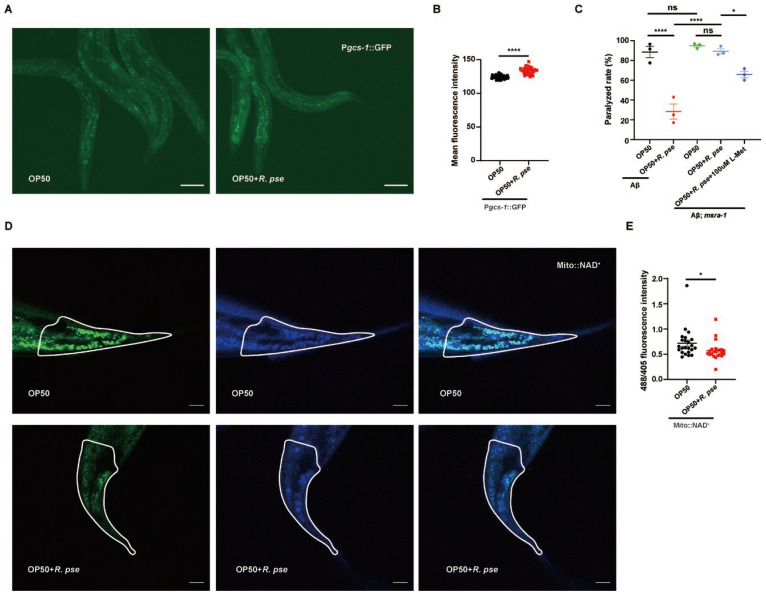
*R. pse* mitigates pathology in the Aβ AD model by anti-oxidative stress. (**A**) Representative fluorescence images of P*gcs-1*::GFP treated with OP50 or OP50 + *R. pse*. Scale bar = 100 μm. (**B**) Quantitative analysis of mean fluorescence intensity of P*gcs-1*::GFP treated with OP50 or OP50 + *R. pse*. Data are the mean ± SEM; two-tailed unpaired Student’s *t*-test. *n* = 32–34. **** *p* < 0.0001. (**C**) Scatter plot showing the paralysis rate of the Aβ AD model or Aβ; *msra-1* treated with OP50 or OP50 + *R. pse*, following L-methionine treatment. One-way ANOVA followed by Tukey’s post hoc test, three independent biological replicates. * *p* < 0.05, **** *p* < 0.0001, ns, not significant (*p* > 0.05). (**D**) Representative fluorescence images of Mito::NAD^+^ treated with OP50 or OP50 + *R. pse*. **Left**, fluorescence image captured under 488 nm excitation; **Middle**, fluorescence image captured under 405 nm excitation; **Right**, merged image of 488 nm and 405 nm. Scale bar = 10 μm. (**E**) Quantitative analysis of the relative fluorescence intensity of Mito::NAD^+^ treated with OP50 or OP50 + *R. pse*. Data are the mean ± SEM; two-tailed unpaired Student’s *t*-test. *n* = 23. * *p* < 0.05.

Next, we sought to elucidate the molecular mechanism underlying the mitigation of AD-related pathology by *R. pse* in the Aβ-based model. Given that oxidative stress serves as a central pathogenic driver in AD [20], we focused on the antioxidative capacity of *R. pse*. GCS-1, which encodes γ-glutamylcysteine synthetase, a key antioxidant enzyme, plays an essential role in oxidative stress resistance [21]. Using a transgenic *C. elegans* strain expressing P*gcs-1*::GFP, we monitored the transcriptional activity of *gcs-1* and found that the *R. pse* treatment significantly increased the GFP fluorescence intensity (Figure 3A,B). This suggests that *R. pse* exerts its neuroprotective effect at least partially by activating the GCS-1-mediated antioxidative stress pathway.

To further verify the antioxidative mechanism of *R. pse*, we employed an *msra-1* mutant strain. MSRA-1 encodes methionine sulfoxide reductase A, a key enzyme responsible for repairing protein oxidation damage [22]. We observed that the *R. pse* treatment no longer delayed paralysis in the *msra-1* mutant AD model, indicating that its neuroprotective effect depends on functional MSRA-1. Methionine sulfoxide reductases are thiol-dependent enzymes that catalyze the reduction of ROS-induced methionine sulfoxide back to methionine [23], and the loss of MSRA-1 thus reduces methionine availability and exacerbates oxidative stress. To further confirm the specificity of MSRA-1-mediated neuroprotection, we supplemented *msra-1*; GMC101 worms with exogenous L-methionine. Methionine supplementation restored the neuroprotective effect of *R. pse* that was abolished in the *msra-1* mutant. Collectively, these results suggest that *R. pse* mitigates AD-related pathology by activating an MSRA-1-dependent pathway to alleviate oxidative stress (Figure 3C).

Nicotinamide adenine dinucleotide (NAD^+^) boosters have been demonstrated to alleviate Aβ proteotoxicity and methylmercury (MeHg)-induced oxidative stress [24,25]. Excessive oxidative stress triggers the hyperactivation of nuclear poly(ADP-ribose) polymerase 1 (PARP-1), and PARP-mediated NAD^+^ depletion has been implicated in the pathogenesis of AD [26]. To determine whether the neuroprotective effects of *R. pse* are associated with elevated cellular NAD^+^ levels, we employed a *C. elegans* strain expressing a mitochondria-targeted NAD^+^ biosensor. This reporter system operates via an inverse fluorescence readout, whereby a decrease in the GFP intensity corresponds to an increase in NAD^+^ availability, allowing for the in vivo quantification of the mitochondrial NAD^+^ status [27]. We measured the relative fluorescence intensity ratio of the 488 nm channel to the 405 nm channel (for normalization) in worms cultured in the presence or absence of *R. pse*. Compared with control animals, the *R. pse*-treated worms exhibited a significantly lower fluorescence ratio (Figure 3D,E), indicating elevated mitochondrial NAD^+^ levels. These findings suggest that the neuroprotective activity of *R. pse* may be mediated, at least in part, through the enhancement of mitochondrial NAD^+^ homeostasis.

### 3.4. R. pse Mitigates Pathology in the Aβ AD Model Through Enhancing Immune Activity

**Figure 4:**
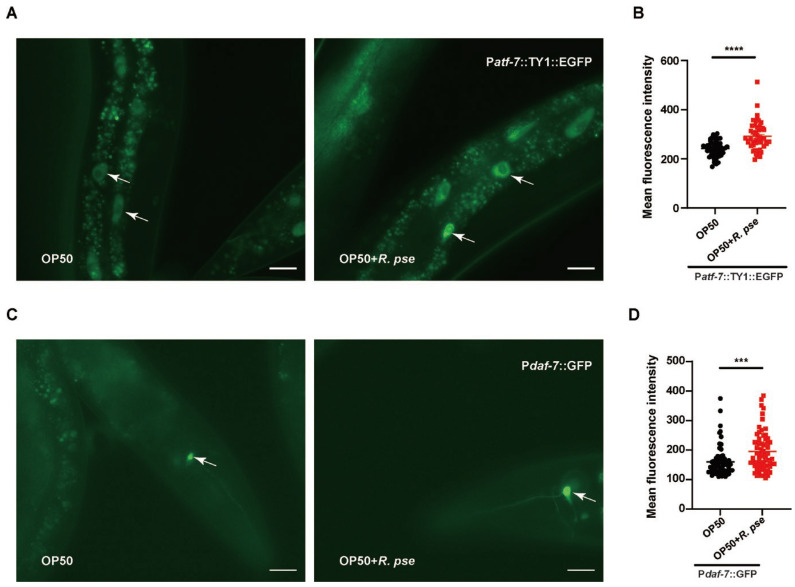
*R. pse* mitigates pathology in the Aβ AD model through enhancing immune activity. (**A**,**B**) Representative fluorescence images and quantitative analysis of fluorescence intensity of P*atf-7*::TY1::EGFP treated with OP50 or OP50 + *R. pse*. Arrows indicate nuclei in intestines. Scale bar = 20 μm. Data are the mean ± SEM; two-tailed unpaired Student’s *t*-test. *n* = 50 (the number of fluorescent intestinal nuclei). **** *p* < 0.0001. (**C**,**D**) Representative fluorescence images and quantitative analysis of P*daf-7*::GFP treated with OP50 or OP50 + *R. pse*. Arrows indicate neurons in heads. Scale bar = 20 μm. Data are the mean ± SEM; two-tailed unpaired Student’s *t*-test. *n* = 66 (the number of fluorescent head neurons). *** *p* < 0.001.

Additionally, mounting evidence has established that the p38 mitogen-activated protein kinase (p38 MAPK)/ATF-7 immune signaling axis mediates neuroprotective functions [28]. To assess the involvement of this pathway in the protective effects of *R. pse*, we quantified the nuclear GFP fluorescence intensity using a knock-in strain expressing GFP-tagged ATF-7. Our results revealed that *R. pse* administration increased the nuclear ATF-7 expression (Figure 4A,B), supporting the activation of the ATF-7-mediated immune signaling cascade.

To further validate the immune-modulatory activity of *R. pse*, we investigated the TGF-β pathway, which is closely linked to innate immune regulation [29]. Using a transgenic reporter strain expressing P*daf-7*::GFP, where *daf-7* encodes a ligand of the TGF-β pathway, we monitored the *daf-7* expression. The fluorescence analysis showed that the *R. pse* treatment elevated P*daf-7*::GFP expression (Figure 4C,D), suggesting that *R. pse* acts through the DAF-7-mediated TGF-β immune pathway.

### 3.5. Untargeted Metabolomic Profiling of R. pse

**Figure 5:**
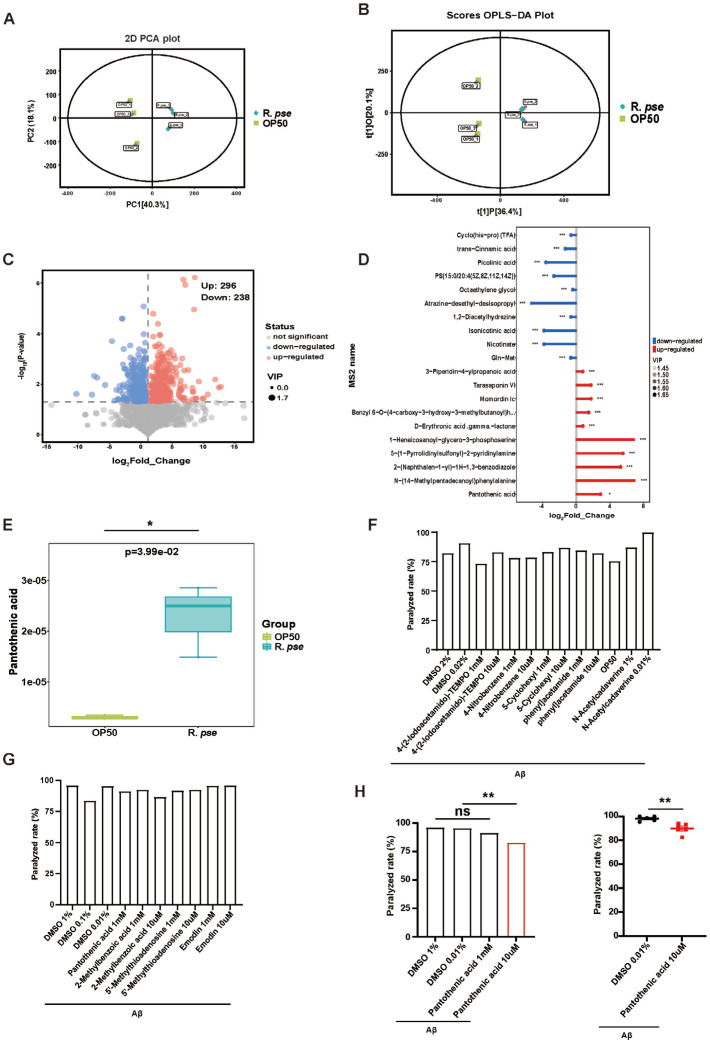
The metabolite pantothenic acid, derived from *R. pse*, mitigates AD phenotypes. (**A**) Score plot of principal component analysis (PCA). (**B**) Orthogonal partial least-squares discriminant analysis (OPLS-DA) plot. (**C**) Volcano plot comparing the OP50 and *R. pse* groups, with red representing upregulated metabolites and green representing downregulated metabolites. (**D**) Matchstick plot of differential metabolites between OP50 and *R. pse* groups. * *p* < 0.05, *** *p* < 0.001. (**E**) Box plot of the relative content of pantothenic acid in OP50 and *R. pse* groups. * *p* < 0.05. (**F**,**G**) Bar graph showing the paralysis rate of the Aβ AD model treated with metabolites of *R. pse*. The chi-square test, *n* > 20 (number of worms subjected to different drug treatments). (**H**) **Left**, bar graph showing screening for effective concentrations of pantothenic acid. The chi-square test, *n* > 20 (number of worms subjected to different drug concentrations). **Right**, paralysis rate of Aβ AD model treated with the control OP50 + 0.01%DMSO or OP50 + 10 μM pantothenic acid. Two-tailed unpaired Student’s *t*-test, five independent biological replicates. ** *p* < 0.01, ns, not significant (*p* > 0.05).

To identify differential metabolites between *R. pse* and the control OP50, we conducted an untargeted metabolomic analysis. The principal component analysis (PCA) demonstrated significant inter-group variability, clearly separating the OP50 and *R. pse* cohorts (Figure 5A), suggesting substantial differences in their global metabolic profiles. The orthogonal partial least-squares discriminant analysis (OPLS-DA) further validated the distinct separation between these groups (Figure 5B). The volcano plot analysis revealed 296 significantly upregulated and 238 downregulated metabolites (Figure 5C). The matchstick plot illustrated the top 10 metabolites with altered expression levels selected based on logFC (fold change), while the radar plot depicted the top 10 differentially expressed metabolites chosen according to *p*-value (Figure 5D).

### 3.6. The Metabolite Pantothenic Acid, Derived from R. pse, Mitigates AD Phenotypes

To identify the functional metabolites derived from *R. pse*, we performed comparative metabolomic screening to identify metabolites that were significantly enriched in *R. pse* compared with the control strain OP50. Among candidate metabolites assessed (Figure 5F,G), pantothenic acid, displaying a 7.4-fold upregulation (Figure 5E), was found to alleviate paralysis in the AD model, with 10 μM identified as an effective concentration through dose–response screening (Figure 5H). These findings suggest that pantothenic acid acts as a bioactive metabolite of *R. pse*.

## 4. Discussion

In the present study, we identified *R. pse* as a beneficial bacterium capable of alleviating pathological deficits in *C. elegans* models of AD, suggesting *R. pse* as a promising microbial candidate for counteracting Aβ-mediated toxicity and cognitive dysfunction in vivo.

Phenotypic characterization revealed that *R. pse* exhibited a comparable growth status and food preference and no adverse effects on brood size or developmental timing relative to the standard laboratory food strain *E. coli* OP50, supporting its biosafety and suitability as a dietary intervention in *C. elegans*. Mechanistically, the neuroprotection conferred by *R. pse* requires viable bacteria or heat-labile cellular components rather than secreted extracellular factors.

Oxidative stress is a well-established core pathogenic mechanism underlying AD progression. Our results demonstrated that *R. pse* upregulated the expression of gcs-1, which encodes a key antioxidant enzyme involved in glutathione biosynthesis, indicating the activation of antioxidant defenses. Moreover, the neuroprotective effect of *R. pse* was abolished in the *msra-1* mutant, which is defective in repairing protein oxidation caused by ROS, and could be restored by exogenous methionine supplementation. These data highlight that the MSRA-1-mediated protein oxidation repair pathway is essential for *R. pse* to mitigate oxidative damage and Aβ-induced toxicity.

Beyond antioxidant signaling, we further revealed that *R. pse* activated innate immune pathways associated with neuroprotection. The *R. pse* treatment increased the nuclear expression of ATF-7, a key transcription factor downstream of the p38 MAPK immune signaling cascade. Additionally, *R. pse* elevated the expression of daf-7, a ligand in the TGF-β immune pathway. These results suggest that *R. pse* exerts neuroprotection not only by enhancing the cellular resistance to oxidative stress, but also by modulating innate immune signaling, which has been closely linked to the clearance of protein aggregates and maintenance of neuronal homeostasis. Nevertheless, our current study only confirmed the activation of the ATF-7 and TGF-β signaling pathways, yet we have not verified through genetic or pharmacological inhibition assays that immune signals are essential for the neuroprotective effects of *R. pse*. Therefore, the activation of immune pathways only shows a correlative relationship, and the causal relationship remains to be confirmed.

Moreover, untargeted metabolomic profiling further revealed distinct metabolic signatures between *R. pse* and OP50, with hundreds of differentially abundant metabolites, implying that specific microbial metabolites may contribute to the observed beneficial effects. Subsequent screening of candidate metabolites identified pantothenic acid as a functional metabolite enriched in *R. pse*, supporting the notion that metabolic components underpin its protective activity against AD-related phenotypes. However, given the limited scope of the screened metabolites, pantothenic acid may only act as a partial mediator of *R. pse* activity rather than the sole effector molecule. Other unselected metabolites may also participate in this biological process.

The limitations of this study should be acknowledged. First, the present study did not directly assess the Aβ aggregation status, oligomer accumulation or its clearance mechanism. It remains unclear whether *R. pse* exerts upstream effects by directly inhibiting Aβ aggregation or functions downstream by enhancing organismal stress resistance. Second, all experiments were conducted exclusively in *C. elegans* models of AD. Due to the restriction of our model and experiment designs, whether *R. pse* can exert sustained neuroprotective effects throughout the aging process remains to be further investigated. Third, although this invertebrate system offers powerful advantages for mechanistic dissection and genetic screening, it lacks the complex brain architecture and adaptive immune responses that characterize the mammalian nervous system. Therefore, the translational relevance of our findings to human AD remains inherently limited. The results presented here should be viewed as hypothesis-generating, and any extrapolation to mammalian pathophysiology warrants caution. Future studies will be necessary to validate the key observations in established mammalian AD models, such as transgenic mouse lines, in order to evaluate the conservation of the identified mechanisms and their potential therapeutic relevance.

In summary, our study demonstrates that *R. pse* mitigates Aβ-induced paralysis and cognitive impairment in *C. elegans* through multiple coordinated mechanisms, including the enhancement of antioxidative stress responses via the GCS-1 and MSRA-1 pathways, and the activation of p38 MAPK/ATF-7 and TGF-β/DAF-7 immune signaling. These findings expand our understanding of the microbial regulation of neurodegenerative pathology and support the potential application of *R. pse* as an intervention for AD-related pathogenesis.

## Figures and Tables

**Table 1:** List of *C. elegans* strains and their characteristics.

Strains	Genotypes	Explanations
N2	Wild type	
GMC101	*dvIs100* [P*unc-54*::A-beta1-42; P*mtl-2*::GFP]	Expresses full-length human Aβ_1-42_ peptide in body wall muscle cells and exhibits a paralysis phenotype
GRU101	*gnaIs1* [P*myo-2*::YFP]	Control strain for pan-neuronal Aβ_1-42_ expressing strain GRU102
GRU102	*gnaIs2* [P*myo-2*::YFP + P*unc-119*:: A-beta1-42]	Pan-neuronal expression of Aβ_1-42_
TV53181	[P*unc-54*::GFP]	Monitoring the expression of *unc-54* promoter
XMW183	*dvIs100*; *msra-1 (tm1421)*	GMC101 with *msra-1* mutant
*wyIs50120*	[P*dpy-7*::cox8 (4x)::NAD^+^ sensor; P*odr-1*::GFP]	Mitochondria NAD^+^ sensor
LD1171	[P*gcs-1*::GFP + *rol-6* (*su1006*)]	Indicator of *gcs-1* expression
FK181	[P*daf-7*::GFP + *rol-6* (*su1006*)]	Indicator of *daf-7* expression
OP638	[P*atf-7*::TY1::EGFP::3xFLAG + *unc-119*(+)]	Indicator of ATF-7 expression

## Data Availability

The original contributions presented in this study are included in the article. The 16S rRNA sequencing results can be viewed at NCBI under the accession number PZ298169. Further inquiries can be directed to the corresponding authors.

## Abbreviations

The following abbreviations are used in this manuscript:

AD	Alzheimer’s disease
ATP	Adenosine triphosphate
GBA	Gut–brain axis
*R. pse*	*Rhodopseudomonas pseudopalustris*
*C. elegans*	*Caenorhabditis elegans*
GCS-1	γ-Glutamylcysteine synthetase
MSRA-1	Methionine sulfoxide reductase A-1
ATF-7	Activating transcription factor 7
TGF-β	Transforming growth factor β
Aβ	Amyloid-beta
MGBA	Microbiota–gut–brain axis
PCA	Principal component analysis
OPLS-DA	Orthogonal partial least-squares discriminant analysis
NAD^+^	Nicotinamide adenine dinucleotide
p38 MAPK	p38 Mitogen-activated protein kinase
